# Altered Plasma Butyrylcholinesterase Activity in Streptozotocin-Induced Diabetic Hypertensive Rats Does Not Reflect Impaired Liver Function

**DOI:** 10.33549/physiolres.935558

**Published:** 2025-06-01

**Authors:** Tibor HODBOD, Kristina SZMICSEKOVA, Aneta CINAKOVA, Kornelia STEFIKOVA, Zora KRIVOSIKOVA, Eva KRALOVA, Anna HRABOVSKA

**Affiliations:** 1 Department of Pharmacology and Toxicology, Faculty of Pharmacy, Comenius University Bratislava, Bratislava, Slovak Republic; 2 Hospital Pharmacy, National Institute for Cardiovascular Diseases, Bratislava, Slovak Republic; 3 Department of Pharmacology, Faculty of Medicine, Slovak Medical University Bratislava, Bratislava, Slovak Republic; 4 Toxicological and Antidoping Center, Faculty of Pharmacy, Comenius University Bratislava, Bratislava, Slovak Republic

**Keywords:** Butyrylcholinesterase, Streptozotocin, Spontaneously hypertensive rats, Diabetes mellitus, Liver damage

## Abstract

Butyrylcholinesterase (BChE) has recently been associated with metabolic imbalance. A correlation between plasma activity and lipid and glucose metabolism has been reported in animal models and human patients. Here, we investigated plasma BChE activity in a rat model of comorbid hypertension and type 1 diabetes mellitus (DM) induced by a single injection of streptozotocin (STZ, 55 mg/kg) in male spontaneously hypertensive rats (SHR) (SHR+DM). The SHR+DM animals exhibit the main characteristics of the human comorbid pathology, including hypertension and hyperglycemia. Although STZ lowered blood pressure in SHR, the animals remained hypertensive as compared to the Wistar controls. Plasma levels of triacylglycerols, cholesterol and HDL were increased, while markers of liver damage such as ALT, AST, were increased and albumin was decreased. Plasma BChE activities were similar in Wistar and SHR. In SHR+DM, plasma BChE activity was increased by 43 %. Interestingly, liver BChE activity and relative mRNA expression were decreased by 60 % in SHR and SHR+DM. While plasma BChE activity is often used as a clinical marker of liver injury, our results suggest that it may not be a reliable indicator.

## Introduction

BChE is an esterase that is ubiquitously and abundantly expressed throughout the body [1,2]. The liver is the main synthetic organ for BChE [3,4], and the highest BChE activities have been reported in the plasma, liver, leg muscle, skin, and brain in both in humans and mice [3,5,6]. Despite this widespread activity, the physiological functions of BChE remain ambiguous. More than 80 mutations have been identified in the human BChE gene, many of which result in loss of enzyme activity or result in the synthesis of a truncated enzyme that is rapidly degraded [3]. Remarkably, both humans with silent BChE and mutant BChE knock-out mice exhibit no clear phenotypic abnormalities [6,7].

An exception to this is an altered response to certain xenobiotics that are typically degraded by BChE. Impaired metabolism in BChE-deficient individuals can lead to serious health complications, such as prolonged apnea following succinylcholine administration [2,8]. It has therefore long been widely accepted that the primary role of BChE in the body is to inactivate xenobiotics containing ester bonds.

However, BChE also plays a critical role in the maintenance of acetylcholine hydrolysis. First, it protects acetylcholinesterase by scavenging organophosphates and carbamates after exposure. Second, BChE acts as a backup enzyme in the breakdown of acetylcholine when acetylcholinesterase is inactivated, as in acetylcho-linesterase-deficient mice [5,9,10].

In a clinical context, plasma BChE activity is often used as a marker of liver function, as its plasma levels are thought to reflect the synthetic capacity of the liver. Indeed, decreased BChE activity is often associated with liver injury, including conditions such as cirrhosis, hepatitis, and liver failure [5,6].

Recent evidence also suggests a role for BChE in energy balance. BChE-deficient mice, although apparently healthy, develop obesity when fed a high-fat diet [7]. This may be related to the ability of BChE to hydrolyze ghrelin at a rate that significantly affects its plasma levels [11,12]. Ghrelin, an orexinergic hormone, has been shown to cross the blood-brain barrier and influence the release of growth hormone from the pituitary gland, thereby affecting appetite, stress response, and insulin sensitivity [12,13].

A link between plasma BChE and metabolic disorders has been reported in human patients. BChE activity is increased in obese patients and correlates with indicators of adiposity, serum lipid markers, such as triglycerides (TAG), low-density lipoprotein cholesterol, high-density lipoprotein cholesterol (HDL), but also with blood glucose, fasting insulin levels, and insulin resistance index [14–19]. Several clinical observations have suggested that BChE may play a role in altered lipoprotein metabolism in hypertriglyceridemia associated with insulin resistance or insulin deficiency in type 2 diabetes mellitus (DM) [14,15]. Similar findings have been reported in rodent models of DM induced by streptozotocin (STZ) application (STZ+DM) [20,21].

In this study, we sought to build upon these findings by analyzing BChE in an animal model of diabetic hypertension, as hypertension is a common comorbidity in patients with diabetes [22,23].

## Methods

### Experimental animals

All experimental procedures were in accordance with the EU Animal Experiments Directive 2010/63/EU for animal experiments and were approved by the Ethics Committee for Animal Experiments of the Faculty of Pharmacy, Comenius University Bratislava and by the State Veterinary and Food Administration of the Slovak Republic (approval numbers Ro-557/11-221/2, Ro-1786/12-221, Ro-1636/17-221). Animals and diets were obtained from the Department of Toxicology and Laboratory Animals Breeding IEPT, CEM SAS (Dobrá Voda, SK CH 240 11). Male Wistar and spontaneously hypertensive rats (SHR), 12 weeks of age, were used in the study. They were kept in an enriched environment and maintained under controlled environmental conditions (22–24 °C, 12 h light/dark cycles) with *ad libitum* access to standard chow diet (consisting of 65 % carbohydrate, 24 % protein and 11 % fat with a total caloric value of 3227 kcal/kg) and water for at least 7 days prior to the start of the experiment. The overall physical health of the animals was observed daily, and body weight was recorded at the beginning and end of the experiment.

### Induction of type 1 diabetes mellitus

After one week of quarantine, SHR were randomly divided into two groups, diabetic (SHR+DM, n=10) or nondiabetic (SHR, n=6). A larger number of animals was assigned to the diabetic group because of the expected higher mortality. Diabetes was induced by a single dose (55 mg/kg) of streptozotocin (STZ, Sigma-Aldrich, Cat. No. S0130) administered by intraperitoneal injection. STZ was dissolved in 0.1 M citrate buffer (Sigma-Aldrich), pH=4.5. STZ administered at doses higher than 40 mg/kg causes beta-cell damage [24,25], thus the rats used in our experiment modeled type 1 DM. Hyperglycemia was confirmed 3 days after STZ administration by measuring the glucose concentration in blood collected from the tail vein under a fasting conditions using the Accutrend® Plus device (Roche®, Switzerland). Only rats with a fasting glucose level of at least 12 mM were included in the experiment. In addition, blood glucose levels were measured *post mortem* at the end of the experiment under non-fasting conditions. This second measurement resulted in higher glucose levels compared to those typically reported *in vivo* levels, likely due to non-fasting conditions and postmortem processes such as glycogenolysis, which can increase glucose levels after death [26]. The control animals comprised naive Wistar rats (n=16) and non-diabetic SHRs described above, which received an injection of saline solution in the same volume as the administered STZ solution. In accordance with the 3Rs of animal research, male Wistar rats of the same age (13 weeks old) were used in this study, which had been ordered for a parallel experiment performed at the department but were not used but were kept under the same conditions as the animals in our experiment, hence the high number of the animals.

### Blood pressure measurement

Systolic arterial blood pressure was measured one day before sacrifice using the tail-cuff method in conscious animals after prewarming the tail to 37 °C. Blood pressure was measured using a noninvasive blood pressure module (NIBP Controller, ADInstruments, Spechbach, Germany) connected to a computer *via* a manometer and a Powerlab 8/30 data acquisition module (ADInstruments). For each data point, five recordings were analyzed, and mean values were calculated. Data analysis was performed using Chart 5 software for Windows (AD Instruments).

### Tissue collection and processing

After 6 weeks, animals were sacrificed by CO_2_ inhalation. Blood was collected from the vena cava inferior and processed to plasma by addition of 8 % Na_2_EDTA×2H_2_O (Sigma-Aldrich, Cat. No. PHR1068) solution and the centrifugation at 1200× g for 15 min at 4 °C. The separated plasma was immediately frozen in liquid nitrogen.

The whole liver was dissected, weighed, and the tissue sample flash frozen in liquid nitrogen. All flash-frozen tissue samples were stored at −80 °C until further analysis. Immediately prior to analysis, frozen livers were homogenized in prechilled, freshly prepared extraction buffer (0.01 M HEPES buffer pH=7.5 (Sigma-Aldrich, Cat. No. H3375), 0.01 M EDTA (Sigma-Aldrich, Cat. No. PHR1068), 0.8 M NaCl (Sigma-Aldrich, Cat. No. 1.06404) and 1 % CHAPS (Sigma-Aldrich, Cat. No. 850500P) in a w/v ratio of 1:5 (100 mg/0.5 ml) using a TissueLyser (Qiagen, Retsch, Germany) machine for 4 min at 25 Hz, then centrifuged at 14000× g for 10 min at 4 °C, and the supernatant was used for later analysis.

### Determination of BChE activity

BChE activity was determined in plasma samples and in freshly prepared liver extracts using 1 mM butyrylthiocholine iodide (Sigma-Aldrich, Cat. No. B3253) as substrate and 0.5 mM 5,5‘di-thiobis-(2-nitrobenzoic acid) (Sigma-Aldrich, Cat. No. D218200) as coloring agent in 5 mM HEPES buffer pH=7.5 by the modified Ellman’s method [27]. The enzymatic activity of the samples was determined in triplicate. The change in absorbance was recorded at 412 nm in a Synergy H4 Hybrid Reader (BioTek, USA). To confirm the reproducibility of our observation, plasma BChE activity was measured repeatedly in a follow-up experiment under the same conditions.

### Assessment of the biochemical parameters

Plasma levels of TAG, total cholesterol, HDL, glucose, albumin, and the liver function enzymes alanine transaminase (ALT) and aspartate transaminase (AST) were determined using an Ortho Clinical Vitros® 250 Chemistry System (Ortho-Clinical Diagnostics, Raritan, NJ, USA) in a certified laboratory at the Department of Clinical and Experimental Pharmacotherapy of Faculty of Medicine, Slovak Medical University, Bratislava.

### RNA isolation and determination of BChE relative expression

The mRNA was isolated from frozen liver tissues using a standard phenol/chloroform extraction method with TRI-Reagent (Sigma-Aldrich, Cat. No. 93289), and then the concentration of mRNA was measured using a Take3 Microvolume plate (BioTek, USA) and a Synergy H4 Hybrid Reader (BioTek, USA). Reverse transcription was performed on a T100™ Thermal Cycler (Bio-Rad, USA) using the High Capacity cDNA Reverse Transcription Kit (Applied Biosystems, USA, Cat. No. 4374966) following the manufacturer’s instructions. RT-qPCR was performed using SYBR Select Master Mix (Applied Biosystems, USA, Cat. No. 4472908) on the StepOnePlus PCR system (Applied Biosystems, USA). The expression of BChE was normalized to the geometric mean of the expression of the housekeeping genes Hprt1 and Actb (Table 1).

### Statistical analysis

Statistical analysis was performed with GraphPad Prism 9.5.1 software using one-way ANOVA followed by Tukey’s *post hoc* analysis to compare differences in observed parameters between experimental groups. Grubb’s test was used to identify potential outliers. Results are presented as mean + SD or mean ± SD. For all analyses, the level of significance was set at p<0.05.

## Results

### Body weight

**Fig. 1:**
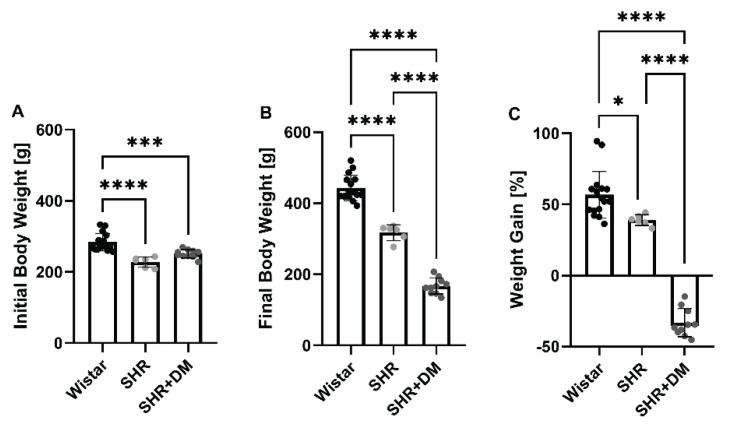
Body weight. Body weight at the beginning of the experiment (**A**), at the end of the experiment (**B**), and weight gain expressed as % weight gain calculated from the initial body weight (**C**). Values are expressed as mean ± SD. * p<0.05, *** p<0.001, **** p<0.0001. SHR, spontaneously hypertensive rats; SHR+DM, strepto-zotocin-induced diabetes in SHR.

SHR and SHR+DM had significantly lower body weights than Wistar rats of the same age both at the beginning (13 weeks of age: 228.00±14.39 g, 251.10±11.77 g, 283.69±24.43 g, respectively, p<0.0001) (Fig. 1A) and also at the end of the experiment (age 18 weeks: 317.00±22.07 g, 167.10±22.44 g, 442.56±35.94 g, respectively, p<0.0001) (Fig. 1B), with SHR+DM having the lowest final body weight. In addition, over the six-week experimental period, SHR had lower percentage increase in the body weight than Wistar rats (56.80±16.38 % vs. 39.02±3.84 %; p=0.0220) (Fig. 1C). Onset and development of diabetes in SHR resulted in a loss of approximately one third of their initial body weight within 6 weeks (Fig. 1C), and their final body weight was 167.10±22.44 g, which was more than 2.5 times lower than the final weight of Wistar rats (p<0.0001) and 1.9 times lower than SHR (p<0.0001) (Fig. 1B).

### Blood pressure

**Fig. 2:**
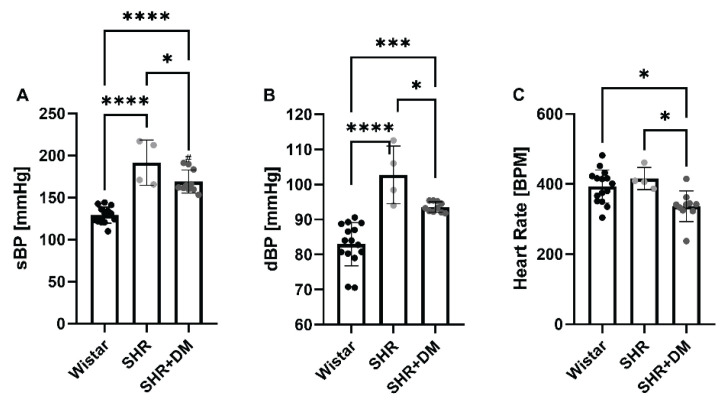
Cardiovascular characteristics. Systolic (**A**) and diastolic (**B**) blood pressure and heart rate (**C**) measured by the tail-cuff method. Values are expressed as mean ± SD. * p<0.05, *** p<0.001, **** p<0.0001. SHR, spontaneously hypertensive rats; SHR+DM, streptozotocin-induced diabetes in SHR; sBP, systolic blood pressure; dBP, diastolic blood pressure; BPM, beats per minute.

As expected, SHR had higher systolic and diastolic blood pressures as compared to Wistar rats (systolic: 191.50±26.95 mm Hg vs. 129.00±9.64 mm Hg, p<0.0001; diastolic: 102.75±8.23 mm Hg vs. and 82.95±6.18 mm Hg, p<0.0001 respectively, Fig. 2A, B). STZ-induced diabetes decreased systolic blood pressure by 12 % (169.0±13.68 mm Hg, p=0.0320 vs. SHR) and diastolic blood pressure by 9 % (93.53±0.44 mm Hg, p=0.0250 vs. SHR), but both remained elevated compared with Wistar rats (p<0.0001 and p=0.0002 respectively). The reduced blood pressure in SHR+DM was accompanied by a reduced heart rate (336±44 BPM, p=0.0120 vs. Wistar and p=0.0155 vs. SHR, Fig. 2C). We did not observe any differences in heart rate between Wistar and SHR groups (393±47 BPM and 416±32 BPM respectively, Fig. 2C).

### Biochemical parameters

Parameters of lipid metabolism and glycemia were evaluated at the end of the experiment (Table 2). While there were no differences in TAG, total cholesterol, HDL, and glucose levels between Wistar rats and SHR, STZ-induced diabetes led to an increase in all these parameters after 6 weeks. Furthermore, plasma BChE activity exhibited a positive correlation with all parameters associated with lipid metabolism (TAG: rs=0.6234, p<0.0001; total cholesterol: rs=0.2233, p=0.0111; HDL: rs=0.3651, p=0.0170). The correlation was also observed between BChE activity and glucose levels (rs=0.3679, p=0.0006).

Biochemical assessment of hepatocyte injury revealed increased AST levels in both SHR and SHR+DM compared to Wistar rats, and increased ALT levels in SHR+DM compared to both SHR and Wistar rats. Plasma albumin levels were comparable between Wistar rats and SHR, but significantly decreased in SHR+DM (Table 2).

### Butyrylcholinesterase activity and expression

**Fig. 3:**
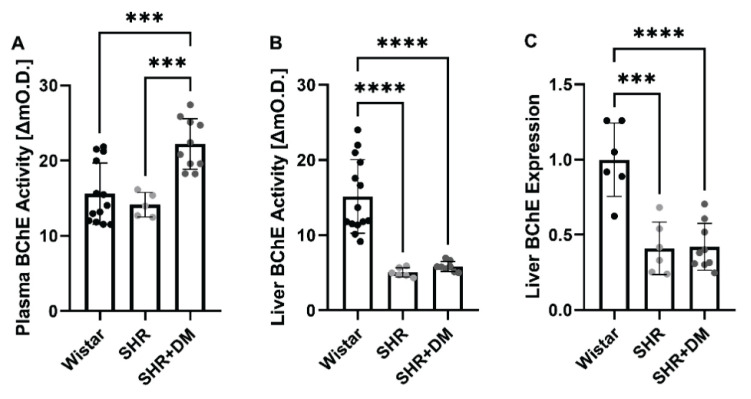
Butyrylcholinesterase activity and relative liver mRNA expression. BChE activity in plasma (**A**), BChE activity in liver extracts (**B**) and relative mRNA expression of BChE in liver (**C**). Values are expressed as mean ± SD. *** p<0.001, **** p<0.0001. SHR, spontaneously hypertensive rats; SHR+DM, streptozotocin induced diabetes in SHR.

Plasma BChE activity was comparable in Wistar rats and SHR (ΔmO.D.: 14.08±4.03 for Wistar, 14.13±1.63 for SHR, Fig. 3A). Surprisingly, STZ-treated SHR showed a 43 % increase in plasma BChE activity (ΔmO.D.: 20.14±5.34, p=0.0004, Fig. 3A). Since the liver is considered to be the major synthetic organ of BChE, the hepatic activity of this enzyme was also evaluated. We observed a significant decrease in BChE activities in the liver of SHR and SHR+DM as compared with Wistar rats, whereas the values of diabetic and non-diabetic SHR were comparable (ΔmO.D.: 15.28±5.07 for Wistar, 5.05±0.62 for SHR and 5.85±0.73 for SHR+DM, p<0.0001, Fig. 3B). This more than 60 % decrease in liver BChE activities was consistent with the approximately 60 % decrease in relative liver BChE mRNA expression. We observed no difference in BChE mRNA expression levels between SHR and SHR+DM (Fig. 3C).

## Discussion

Here, we present our data from SHR+DM, an animal model of comorbid DM and hypertension. Hypertension is a common comorbidity of DM, observed in 20–50 % of patients worldwide [22,28–31]. The comorbidity of both pathologies has a synergistic effect on the development of cardiovascular disease, increasing the morbidity and mortality of the patients [32]. In our experiment, markers of cardiovascular pathology associated with cardiovascular mortality were affected in SHR+DM. STZ-induced diabetes in SHR increased the levels of TAG and total cholesterol. A mild to moderate increase in TAG levels, even after adjustment for HDL, increases the risk of coronary heart disease, myocardial infarction, and mortality [33,34], while hypertriglyce-ridemia is common in uncontrolled DM. On the other hand, HDL, which enables the transport of cholesterol from blood and peripheral tissues to the liver and thus high levels are considered cardioprotective, was slightly increased in SHR after STZ injection.

STZ-induced diabetes resulted in a slight decrease in both systolic and diastolic blood pressure, but remained significantly higher as compared to Wistar rats. This is in agreement with the results of Somani *et al*. [35] and Susic *et al*. [36] who also observed a hypotensive effect. In addition, the hypertension in SHR+DM was accompanied by a slight bradycardic effect, a finding also reported by Susic *et al*. [36]. As no such effect was observed in SHR, we speculate that decreased heart rate may be a sign of myocardiopathy as a result of the synergistic effect of comorbidities.

Hypertensive patients have increased BChE activities as compared to normotensive subjects [37]. BChE may contribute to blood pressure regulation *via* T cell-derived acetylcholine. Acetylcholine reduces vascular tone after binding to the endothelium-localized muscarinic receptors *via* activation of the nitric oxide (NO) pathway [38–41]. Impaired degradation of acetylcholine by altered BChE activity or inhibited NO synthesis may therefore affect the ability of vascular smooth muscle cells to relax, which is reflected in the blood pressure response. However, hypertension is usually present in polymorbid patients suffering from other pathologies such as obesity, DM or even metabolic syndrome, which are also known to influence serum BChE, making a causal relationship with hypertension difficult to establish. Increased BChE activity has also been observed in animal models of hypertension induced by Nω-Nitro-L-arginine methyl ester hydrochloride (L-NAME) [42]. Although we did not observe a change in plasma BChE activity in SHR, we have previously shown that BChE activity in SHR aorta is decreased in a segment-specific manner compared with Wistar aorta, with a drastic decrease in the aortic arch, a mild decrease in the thoracic aorta, and a trend toward a decrease in the abdominal aorta [43]. The discrepancy between our results from SHR and those from L-NAME-induced hypertension could be explained by the different mode of pathogenesis. Whereas hypertension develops spontaneously in SHR due to polygenic origin [44], L-NAME is a NO synthase inhibitor that causes endothelium-dependent contraction [45–47]. Thus, different regulatory mechanisms are altered in these animal models of hypertension.

In contrast to SHR, plasma BChE activity was increased in SHR+DM. This change in activity could be attributed to several characteristics of this rat model. First, an impaired lipid metabolism could be considered. Indeed, a relationship between BChE activity and lipid metabolism has been proposed by many authors. In BChE-deficient mice treated with a high-fat diet, increased serum total cholesterol and hepatic TAG levels were associated with hepatic steatosis [48]. Adeno-associated virus gene transfer of mouse BChE resulted in decreased liver weight and TAG levels [48]. Also, pharmacological reduction of BChE activity in rats, such as by dexamethasone, significantly increased TAG and total cholesterol levels [49]. Similarly, in our experiment, we observed increased levels of TAG and total cholesterol in SHR+DM compared to SHR and Wistar. In addition, a positive correlation was observed between plasma BChE activity and the lipid markers studied. Notably, a correlation between serum BChE activity and TAG levels has also been shown in human patients with type 1 DM [50] and type 2 DM [20]. Previous studies have also reported correlations between insulin levels, blood glucose, and BChE activity in DM patients, suggesting that the enzyme may be modulated by glycemic control and insulin sensitivity [15,50]. These reports are consistent with our results, as a positive correlation was observed between plasma BChE activity and glucose levels.

Another biochemical marker affected by STZ injection in SHR was serum albumin, the main marker of visceral protein depletion. Decreased albumin levels could indicate malnutrition, which is a possible explanation considering the decreased body weight in SHR+DM. However, in contrast to our results of increased serum BChE activity, other authors report decreased BChE activity in malnourished patients [51,52]. Decreased albumin levels are consistent with increased liver markers, ALT and AST, strongly suggesting liver damage. Also, plasma/serum BChE is considered by some clinicians to be a marker of impaired liver function [52], as the liver is considered to be the source of soluble forms of BChE in the blood. Indeed, we observed decreased BChE activity and mRNA expression in the liver. However, as discussed above, plasma BChE activity was increased in SHR+DM. Thus, based on our results, plasma BChE activity is not a reliable indicator of liver damage and should not be used as a clinical marker for diagnostic purposes.

In summary, the SHR+DM animal model exhibits numerous characteristics associated with comorbid DM and hypertension in humans, including impaired glucose and lipid metabolism and altered hemodynamic parameters. STZ-induced diabetes in hypertensive rats resulted in increased levels of TAG and total cholesterol, both of which are associated with cardiovascular risk. Although STZ slightly lowered blood pressure, the hypertensive state persisted with accompanying bradycardia, possibly indicating under-lying myocardiopathy.

Increased plasma BChE activity observed in SHR+DM suggests a potential involvement of this enzyme in the pathophysiology of concomitant diabetes and hypertension, possibly through its association with lipid metabolism. However, despite the observed decrease in albumin levels and indications of liver damage, plasma BChE activity did not serve as a reliable marker of liver function in this model. These findings underscore the complexity of BChE regulation in the context of comorbid conditions and highlight the need for further research to clarify its clinical relevance in metabolic and cardiovascular disorders.

## Figures and Tables

**Table 1:** Forward (F) and reverse (R) primer sequences (5′ - 3′).

*Gene*		Sequence
*Bche*	F	GGGCTGAGGAAATCTTTAGTCGA
	R	GGAGCCCGGAGTTTAGAGTTTA
*Hprt1*	F	CAGCTTCCTCCTCAGACCGCTTT
	R	TCACTAATCACGACGCTGGGACTG
*Actb*	F	CCGCGAGTACAACCTTCTTG
	R	GCAGCGATATCGTCATCCA

**Table 2:** Biochemical parameters.

*Parameter*	Wistar	SHR	SHR+DM	p values	
				*	^#^
*TAG (mmol/l)*	1.37 ± 0.68	0.87 ± 0.19	4.23 ± 1.30^****^,^####^	<0.0001	<0.0001
*Total cholesterol (mmol/l)*	1.77 ± 0.40	1.59 ± 0.16	2.20 ± 0.41^*^,^##^	0.0202	0.0097
*HDL (mmol/l)*	1.13 ± 0.22	1.08 ± 0.09	1.45 ± 0.30^*^,^#^	0.0210	0.0211
*Glucose (mmol/l)*	10.52 ± 5.03	15.04 ± 4.06	24.44 ± 3.72^****^,^###^	<0.0001	0.0010
*ALT (μkat/l)*	0.51 ± 0.17	0.56 ± 0.06	1.05 ± 0.40^***^,^##^	0.0004	0.0043
*AST (μkat/l)*	1.35 ± 0.35	2.23 ± 0.44^**^	2.30 ± 0.62^**^	0.0022/0.0010	
*Albumin (g/l)*	32.34 ± 2.82	34.16 ± 2.33	25.10 ± 2.47^****^,^####^	<0.0001	<0.0001

Values are reported as mean ± SD; p value (^*^) vs. Wistar, p value (^#^) vs. SHR. TAG, triacylglycerols; HDL, high density lipoprotein; ALT, alanine transaminase; AST, aspartate transaminase; SHR, spontaneously hypertensive rats; SHR+DM, streptozotocin induced diabetes in SHR.
